# The Influence of the Thermal Treatment of Copper Slag on the Microstructure and Performance of Phosphate Cements

**DOI:** 10.3390/ma16186249

**Published:** 2023-09-17

**Authors:** Rania Derouiche, Patrick Ninla Lemougna, Guillermo Meza Hernandez, Jun Gu, Samir Baklouti, Hubert Rahier

**Affiliations:** 1 Department of Materials and Chemistry, Vrije Universiteit Brussel, Pleinlaan 2, 1050 Brussels, Belgium; lemougna@yahoo.fr (P.N.L.); guillermo.meza.hernandez@vub.be (G.M.H.); jun.gu@vub.be (J.G.); hubert.rahier@vub.be (H.R.); 2 Laboratory of Advanced Materials, National School of Engineers of Sfax, University of Sfax, Sfax 3038, Tunisia; samir.baklouti@fss.usf.tn; 3 Strategic Initiative Materials in Flanders (SIM), 9052 Zwijnaarde, Belgium; 4 Department of Minerals Engineering, School of Chemical Engineering and Mineral Industries (EGCIM), University of Ngaoundere, Ngaoundere P.O. Box 454, Cameroon

**Keywords:** copper slag, thermal treatment, fayalite, acid-activated, phosphate cement

## Abstract

In general, phosphate cements have a very rapid setting reaction at room temperature. The same holds for copper slag-based phosphate cements. This means that using them as a binder, for instance as mortar, is always possible on a small scale, but very difficult on a large scale. In this paper, the heat treatment of the copper slag was shown to be an effective way to increase the setting time and keep the mix workable for an adequate period. The main objective of this research was to examine the changes in the phase composition of quenched copper slag after exposure to 500 °C and to evaluate the impact of these changes on the reactivity of the material in an acidic environment, as well as on the mechanical properties, microstructure, and structure of the produced phosphate cement materials. Various experimental methods were utilized to characterize the raw materials and the obtained phosphate cementitious materials, including isothermal microcalorimetry (TAM Air), thermogravimetric analysis (TGA), infrared spectroscopy (FTIR), X-ray diffraction (XRD), scanning electron microscopy (SEM), as well as the determination of the chemical composition using X-ray fluorescence (XRF) and the particle size distribution. Furthermore, compressive strength tests were conducted to gauge the mechanical resistance of the materials. The main findings of this work revealed that subjecting the copper slag to a thermal treatment of 500 °C induced a partial transformation in its structure. The high temperature caused the oxidation of some of the divalent iron oxide in the slag, leading to the formation of hematite. This treatment increased the setting time and reduced the reactivity of the copper slag with phosphoric acid, ultimately enabling the production of a dense phosphate-based cementitious material with outstanding mechanical properties. The compressive strength of the newly developed cement was recorded to be greater than 78.9 MPa after 7 days, and this strength continued to increase, reaching 82.5 MPa after 28 days.

## 1. Introduction

During the last decades, there has been a growing emphasis on the utilization of inorganic polymers to transform industrial waste streams into construction materials and reduce carbon dioxide emissions. In fact, inorganic polymers are considered a class of green cements [1,2]. Wilson and Nicholson [3] were the first to introduce phosphate bonded cements, categorizing them as acid–base cements (ABCs). They extensively studied phosphate cements that were used as dental cements, as all the compositions they used set within minutes of mixing. The formation of a cement is based on the dissolution of bi or trivalent cations from a precursor followed by the precipitation of the cation phosphate. In the field of construction materials, considerable research has been performed on binders based on an aluminosilicate precursor with an activator solution under appropriate temperature conditions [4]. In contrast, the use of iron-rich slag as a precursor is rather limited. Previous state-of-the-art work demonstrated that phosphate-based cements based on aluminosilicates were characterized by outstanding properties, such as an excellent mechanical strength and good dielectric properties [5,6]. In addition, an exceptional thermal stability of phosphate-based cements was observed with no sign of melting up to 1550 °C [7]. These properties were influenced by several factors, as discussed by Celerier et al. [8,9]. Among the most prominent ones, we noted the choice of the precursor, as it can directly influence the formation, structure, and properties of phosphate cements. A study conducted by A.S. Wagh [10] examined the formation of chemically bonded phosphate cements by slowly stirring mono or divalent oxides that showed a sparse solubility into a phosphate solution. When the solubility of the oxides was low, there was an insufficiency of ions, which hampered the reorganization into a three-dimensional framework. Conversely, a higher solubility led to rapid crystalline precipitation. In addition, Djobo et al. [11] conducted a study on the production of phosphate-based cements using volcanic ash as a precursor. The findings of the study suggested that iron (Fe^2+^) was the primary element that reacted with phosphoric acid, followed by aluminum, calcium, and magnesium. In contrast, silica was deemed inert in relation to this acid. Subsequently, Bewa et al. [12] studied the effects of amorphous silica, iron oxide, and quartz in the raw aluminosilicate precursor on the compressive strength and microstructural properties of acid-based cements. Three different metakaolins, along with waste-fired brick, were utilized in the production of the geopolymers. As the availability of raw material resources [13,14] becomes more and more limited, the need to find alternative precursors based on waste or industrial by-products intensifies. For ecological reasons, the valorization of these by-products can reduce the huge amount of produced slag, which remains unutilized.

The global copper industry produces large amounts of waste that is usually discarded in landfills, causing severe environmental problems [15]. Therefore, the utilization of slag generated during metal production holds significant advantages. Not only does it contribute to environmental protection, but it can also be repurposed as a valuable feedstock in the synthesis and creation of new useful materials. In line with the current best available techniques in pyrometallurgical copper production, the copper slag generated during the smelting and converting process undergoes flotation de-coppering. The phase composition is affected by the cooling conditions following the pyrometallurgical process. Fayalitic slags produced during this process can be quenched to obtain a significant fraction of the amorphous phase [16]. The chemical composition of slag is closely tied to the raw materials that are employed and the overall process, including the type of furnace that is used [16]. Consequently, the chemical composition and mineralogy of slag can vary based on its place of origin and the feedstock [16]. This variability can have significant implications on the properties of the resulting synthesized material, as it may be influenced by changes in the characteristics of the slag [17]. With this in mind, geopolymer technology is focused on industrial waste utilization.

The valorization of industrial by-products has been of increasing interest in the scientific community [18,19]. Although the alkali activation of copper slag has been extensively researched [20,21], the potential for acidic activation has received little attention. This scarcity of research and documentation underscores the potential for further exploration and investigation in this area. During the early 2000s, Wagh [22] conducted research to explore the potential of employing iron oxides for the synthesis of iron phosphate cements. The study revealed that both FeO and Fe_3_O_4_ could readily react with phosphoric acid or acid phosphate solutions at room temperature, yielding IPC. In contrast, Fe_2_O_3_ couldn’t be directly employed to produce IPC through reactions with any phosphate solution, primarily due to its limited solubility even in an acidic environment. Wei et al. [23] also recently presented findings regarding the potential to fabricate iron phosphate cement by employing Fe_3_O_4_ to react with a phosphoric acid solution. Further research undertaken by Luo et al. [24] and Zhang et al. [25] explored the feasibility of producing phosphate cements using industrial solid waste that is rich in silicate minerals. Subsequently, Luo et al. [26] delved into the utilization of iron silicate-rich copper smelting slag to synthesize a novel acid–base cement, referred to as ferrous oxalate cement, through a reaction with oxalic acid. Their work revealed that the iron compounds present in copper slag, including fayalite and magnetite, could readily engage in a reaction with oxalic acid at room temperature. The same group of authors [27] recently explored the feasibility of producing iron phosphate cement using copper slag (CS) as the source of iron and ammonium dihydrogen phosphate (ADP) as the acidic phosphate, which can react at room temperature. Their investigations revealed that the hydration products primarily included iron dihydrogen triphosphate hydrate (FeH_2_P_3_O_10_⋅H_2_O), iron (III) phosphate (FePO_4_), and a small proportion of amorphous phases. These products were formed through the reaction between ADP and the iron-containing phases present in CS, such as fayalite and magnetite. In addition, Katsiki et al. [28], Nikolov et al. [15], and Derouiche et al. [29] researched the acid activation of fayalite slag with phosphoric acid. These authors found that the reaction of raw copper slag activated by phosphoric acid was rapid. It resulted in a solid ferro-phosphate cement within minutes, which was too quick to be useful as a construction material.

The goal of this work was to reduce the reactivity of copper slag, allowing it to set only after a few hours at room temperature, while at the same time obtaining a phosphate cement with decent mechanical properties that was at least comparable to CEM I. Inspired by the findings in the literature, we proposed to study the effects of the oxidation of copper slag to reduce its reactivity with phosphoric acid. A rapid oxidation could be achieved by simply using heat treatment.

Hence, the motivation for the present work was, firstly, to heat treat the copper slag at 500 °C in order to partially crystalize it, prolong the setting time, and ensure sufficient time for homogenization during the mixing, handling, or casting of the copper slag and the phosphoric acid mixture. The reactivity of the fresh phosphate cementitious mixtures was characterized using isothermal differential scanning calorimetry. In the second step, the characterization of the copper slag used before and after thermal treatment was carried out in order to better understand the starting material in terms of its composition, structure, and behavior with respect to the heating temperature, and therefore, to gain a more comprehensive understanding of the structure of the phosphate cement. An X-ray powder diffraction analysis, infrared spectroscopy, and scanning electron microscopy were used to characterize the raw material CS before and after heat treatment at 500 °C (CS and CS500), as well as the obtained phosphate cement materials. In the third step, the compressive strength and Young’s modulus of the obtained phosphate cements were tested after different curing times. In this study, the term phosphate cements will be used to describe the prepared material through the acid activation of the heated copper slag.

## 2. Materials and Methods

**Figure 1:**
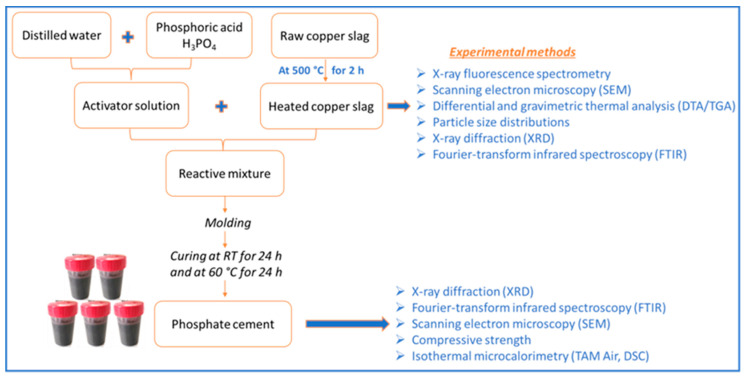
Flowchart of the experimental protocols and adopted methods.

A summary of the flowchart of the experimental protocol and the adopted methods is shown in Figure 1.

### 2.1. Materials

The materials used to prepare the inorganic phosphate cementitious specimens included copper slag (CS), which was kindly provided by Aurubis Belgium. The CS was received in milled form with a Blaine surface area of 2600 cm^2^/g. The Blaine surface value was determined using an air permeability apparatus following the method described in EN 196-6:2018 [30]. The chemical composition of the CS was analyzed using X-ray fluorescence (PW 2400 Philips; MTM, KU, Leuven, Belgium) on the powder (Table 1). It was used without any further modifications. The chemical composition was mainly determined by the dominance of iron oxide and silicon dioxide, constituting up to 80% of its content. The iron was mainly in the Fe (II) form and, to a minor extent, as Fe (III) in the silicate and oxide phases. A wide variety of other elements was also present at low concentrations. Phosphoric acid (commercial H_3_PO_4_ 85 wt% from VWR Chemicals) was used to prepare the activating solution with the addition of distilled water.

### 2.2. Samples Preparation

**Figure 2:**
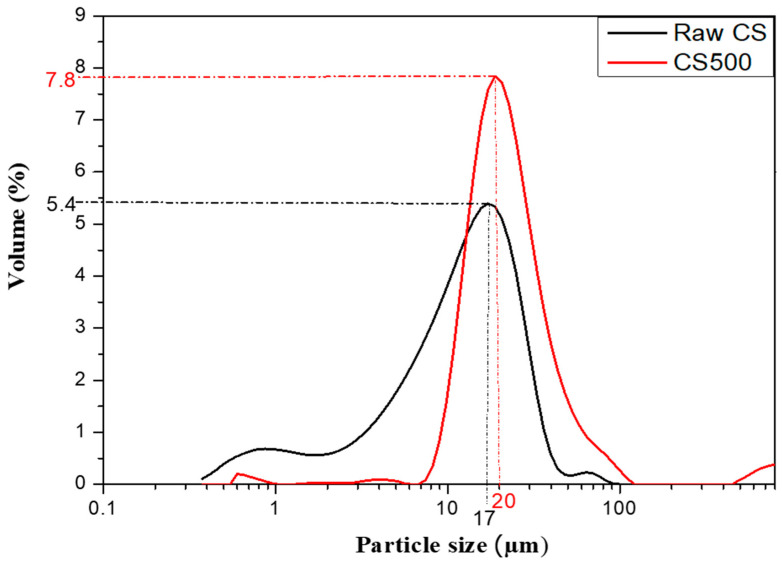
Particle size distribution of the CS and CS500 samples.

The heat treatment was performed by heating the copper slag (CS) at a temperature between 300 °C and 700 °C (in steps of 100 °C) for 2 h in a programmable electric furnace using a heating rate of 5 °C/min. Based on these experiments and on the simultaneous differential thermal and thermogravimetric analyses (DSC–TGA, see results and discussion), the treatment at 500 °C for 2 h was selected as the heating regime for the copper slag, giving an appropriate setting time for the synthetized phosphoric cement. More information on the effect of the treatment temperature on the properties will be provided in an upcoming paper. The heated copper slag (CS500) was crushed manually using a ceramic mold to reduce agglomeration until a fine powder was obtained with a comparable particle size distribution as the initial CS. The particle size distribution of the CS and CS500 samples is presented in Figure 2. The raw CS curve revealed a bimodal granular distribution, which indicated the presence of two types of populations. In contrast, CS500 had a single population and a monomodal distribution.

The phosphate cementitious mixtures were prepared by thermally mixing the treated copper slag CS500 with a phosphoric acid solution. For a fixed quantity of CS500, an appropriate volume of phosphoric acid was added to obtain an (Al+Fe)/P molar ratio equal to 1.5. This ratio was fixed since it gave the phosphoric a cement with the highest mechanical resistance, according to the preliminary studies. The phosphoric acid was mixed with the minimum required amount of distilled water to achieve the desired workability of the mixture with a water-to-solid mass ratio (W/S) equal to 0.12. The fresh mixture was homogenized for 2 min using a kitchen aid, poured into cylindrical plastic molds (60 mm × 32 mm), and manually vibrated to expel the air bubbles. The specimens were sealed to prevent the evaporation of the water and were stored for 24 h at room temperature. The samples started to set at that moment but were too weak be demolded. A post-cure for 24 h at 60 °C was done to speed up the hardening. Then, the specimens were demolded and kept at room temperature for 7, 14, and 28 days before being characterized by the different techniques.

The phosphoric cementitious samples were labeled as PC^a^, where (a) refers to the corresponding time at room temperature before testing.

### 2.3. Characterization Methods

The particle size distribution (PSD) of the raw materials (raw CS and CS500) was measured using laser diffraction ((LS 13 320, Beckman Coulter, Brea, CA, USA) at the department of Materials Engineering (MTM, KU, Leuven, Belgium)) on the powders.

Sample fragments were used to perform the characterization of the microstructure and the interphase using a Phenom Pro Desktop scanning electron microscope (SEM, Philips, Amsterdam, The Netherlands) at an accelerating voltage of 5 kV. The fragments were coated by carbon prior to measurement.

The DSC experiments were performed on a Mettler Toledo DSC822e device (Columbus, OH, USA) with a 500/FRSS/ID502 cell. Nitrogen (N_2_, 100 mL/min) was used as a purge gas. The calibration of the instrument was carried out using indium and zinc. The measurements were performed by subjecting the fresh pastes (approximately 20 mg of the sample) to a temperature scan and by using stainless steel, reusable, high-pressure crucibles. In order to determine the optimum curing temperature for the cement sample, two curing temperatures were examined: room temperature (20 °C) and 60 °C.

Isothermal (20 °C) calorimetry (TAM Air, TA instruments, 159 Lukens Drive, New Castle, DE, USA) was performed on 10 g of the solid part plus the added phosphoric acid solution, according to the L/S mass ratio. The mixture was prepared in an ampoule outside the calorimeter and then transferred into the calorimeter after being mixed for 2 min at 1600 rpm. Data collection commenced 45 min after the mixing process began.

The thermogravimetric analysis (TGA) was performed using the SDT Q600 technology from TA Instruments, Dallas, TX, USA. The analysis involved heating the sample from room temperature to 1000 °C at a heating rate of 10 °C/min under a nitrogen flux. Approximately 13 mg of the sample was used for the measurements.

The mineral phases were determined through the X-ray diffraction measurements using a D2 diffractometer from Bruker Nederland BV, Leiderdorp, The Netherlands. CuKα radiation was used with an acceleration voltage of 30 kV and a current of 10 mA. The measurement involved a step size of 0.020° and a °2θ range spanning from 10° to 70°. The phase identification was performed using the HighScore plus (3.0) software.

Fourier transform infrared spectroscopy (FTIR) was performed using a Nicolet 6700 FT-IR spectrometer from Thermo Fischer Scientific, Waltham, MA, USA. The analysis was carried out on KBr pellets. For each sample, 32 scans were recorded with a resolution of 4 cm^−1^ in a frequency range from 4000–400 cm^−1^.

The elastic modulus test was performed using an ultrasonic pulse analyzer (Pulsonic, Controls, Via Salvo D’Acquisto, 2 Liscate, Milan, Italy). The purpose of this test was to measure the transit time of the ultrasonic pulses through the samples. By knowing the dimensions of the specimens, it was possible to estimate the pulse velocity and modulus of elasticity.

The compressive test was performed using an Instron 5885H (Norwood, MA, USA) with a maximum load of 250 kN. The displacement rate of the machine’s head was set to 1 mm/min until the sample failed. For each test, three replicates were performed under the same testing conditions. All the reported values for the mechanical strength were calculated as the average of the measurements from these three tests and expressed in MPa. The error bar represents the standard deviation obtained from the replicate specimens.

In this section, we presented the raw materials that were used, the chosen nomenclature, and the adopted protocols. In addition, we exposed the principles of the different characterization techniques that were used in order to achieve the research objectives.

## 3. Results

### 3.1. Temperature Induced Transitions of the Raw Copper Slag

**Figure 3:**
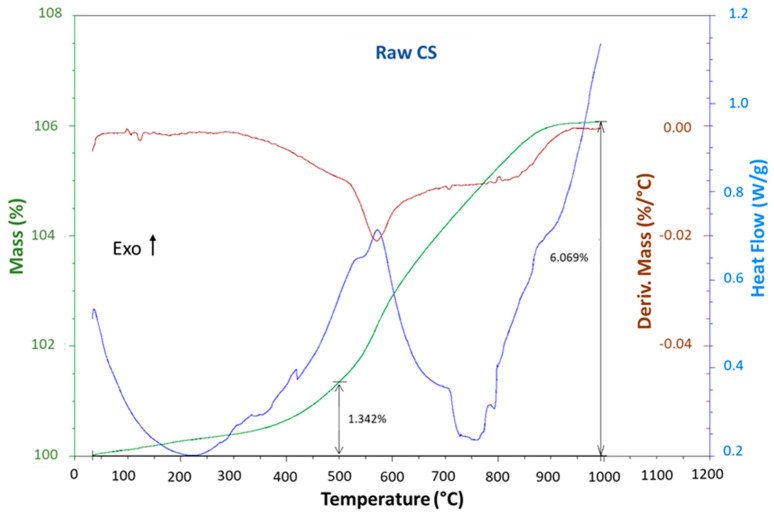
DSCTGA curves of the raw copper slag. The oxidation of the CS increased between 400 °C and 900 °C, as seen from the mass increase and exothermic signal.

The thermal transitions of the raw copper slag were assessed using DSC–TGA (Figure 3). It revealed the presence of an exothermic effect that coincided with a progressive increase in the mass of the slag in the temperature range from 400 to 900 °C, which was due to slag oxidation [31]. This finding was in total agreement with Mihailova et al. [32] and Gradinarov et al. [33], who reported that the marked sample mass increase in the TGA signal with the increase in temperature up to 1000 °C was due to the oxidation processes of the copper slag (mainly from Fe^2+^ to Fe^3+^). The 6.07% increase in the mass, compared to the mass of the Fe^2+^ present according to XRF (Table 1), proved that all the Fe^2+^ had been transformed into Fe^3+^. This process passed through a four-stage mechanism, which was accompanied by the structural transformation and structural formation of ordered plate-like crystals of hematite and an amorphous silicate phase, according to the following reaction.

2FeO·SiO_2_ (Fayalite) + 1/2 O_2_→ α-Fe_2_O_3_ (Hematite) + SiO_2_ (amorphous silica) (1)

Additionally, in the adjoining layer, unoxidized fayalite, magnetite (Fe_3_O_4_), and silica were formed, as was rightly explained by Marangoni et al. [34], according to the following reaction.

2F_e_O·SiO_2_ (Fayalite) + 1/2 O_2_ → 2/3 Fe_3_O_4_ (Magnetite) + SiO_2_ (amorphous silica) (2)

The objective of this study was to perform a partial oxidation of the copper slag, for which the selected temperature was 500 °C. At this specific temperature, the thermogravimetric analysis (TGA) curve exhibited a mass increase of 1.34%, confirming the oxidation of 26% of FeO into Fe_2_O_3_. With the heat treatment conditions applied in this study (exposure to 500 °C for 2 h), the mass increased by approx. 4%, as measured via a separate measurement, proving that 77% of the Fe^2+^ had been converted into Fe^3+^. It is, however, possible that other oxidations occurred, as for only Fe^2+^ to oxidize into Fe^3+^, an increase of only 5.2 wt% instead of 6.07 was expected. It was known that a very small amount of Fe was also present that was also oxidized, which could explain the extra oxidation. The increase in the mass did not happen in one smooth step. Instead, it started at room temperature (very slowly) and in the region of 400 °C to 800 °C, where at least two steps could be distinguished. The oxidation of the slag and its effect on the formation of phosphate cement will be studied in more detail in an upcoming paper.

In conclusion, the oxidation for 2 h at 500 °C transformed part of the Fe^2+^ into Fe^3+^. The effect on the phase transformations in the slag will be studied in the next section. Additionally, the phases present in the final phosphate cement will be investigated.

### 3.2. XRD Analysis of the CS, CS500, and PC Samples

**Figure 4:**
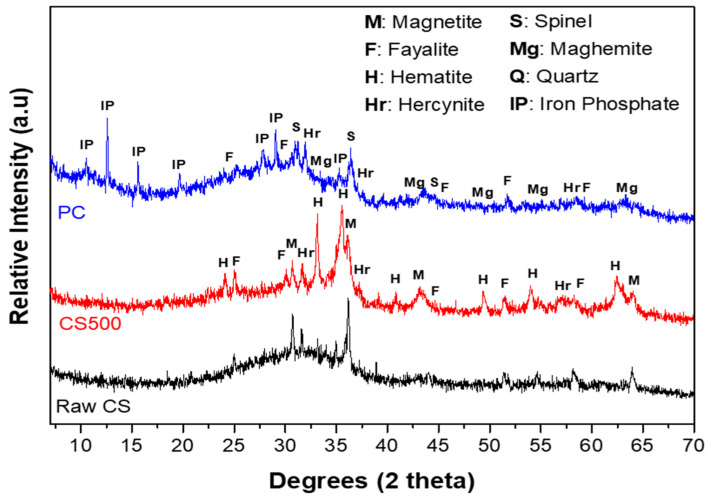
XRD patterns of the raw CS, CS500, and PC samples after 28 curing days.

The X-ray patterns of the raw CS, CS500, and PC are presented in Figure 4. From the depicted results, the raw CS was mainly an amorphous material with few crystalline features, mainly ascribed to fayalite (Fe_2_SiO_4_) and magnetite (Fe_3_O_4_). The diffuse halo located between 25 and 40° 2θ was related to the amorphous silicate and iron phases. This result was in line with Miltiadis et al. [35], who reported that quenched CS was at least 95% amorphous. Other authors such as Nikolov et al. [15] reported that the amorphous phase in the raw copper slag was rich in divalent ions (Fe^2+^) present in the structure of fayalite (Fe_2_SiO_4_) and magnetite (Fe_3_O_4_). As discussed by Wagh et al. [10], most of the divalent oxides were sparsely soluble, and they could be readily reacted with a phosphoric acid solution to form a solid material. These findings explained the fast setting of the raw copper slag in an acidic medium.

The thermal treatment at 500 °C of the copper slag caused the appearance of crystalline features that were mainly ascribed to hematite (Fe_2_O_3_) and hercynite (FeAl_2_O_4_) as a result of the partial oxidation of fayalite at 500 °C, as predicted by Equation (1) (Section 3.1), and the presence of some diffractions corresponding to fayalite and magnetite that were already present, although mainly in the amorphous form. This oxidation of Fe (II) was already observed in TGA. Apart from the crystalline phases, the diffuse halo located between 30 and 40° 2θ, linked to the amorphous fraction of the CS500 structure, shrunk compared to the raw CS. The slight displacement and decrease in the diffuse halo centered at 32° 2θ in the raw CS sample to 35° 2θ in the CS500 indicated a change in the nature of the amorphous phase and the presence of the lower amorphous phase content in the structure of the CS500. The hematite reflection peaks observed at 33.1° and 34.7° 2θ were broad, corresponding to nanocrystalline hematite. These broad peaks indicated the presence of amorphous iron oxide in the structure. Obviously, heating the slag at 500 °C resulted in a partial transformation of the divalent ions (Fe^2+^) from magnetite (Fe_3_O_4_) and fayalite (Fe_2_SiO_4_) to trivalent ions (Fe^3+^) in the form of hematite (Fe_2_O_3_). This was explained by Rabadjieva et al. [36], who proved that the oxidation and decomposition of the fayalite and magnetite into hematite took place simultaneously. The thermal treatment, thus, caused a significant decrease in the amorphous content of the copper slag but not a total crystallization under these conditions.

The third observation was made on the XRD pattern of the CS500-based phosphate cementitious material (PC). The analysis of this diffractogram revealed the appearance of the characteristic peaks of the crystallized phases (iron phosphate, spinel, quartz, and maghemite) with the presence of two diffuse halos characteristic of an amorphous material. The characteristic peaks of hematite previously observed at 33.1 and 35.6° 2θ on the XRD pattern of CS500 disappeared on the XRD patterns of the phosphate-based cements (PC). This implied that the hematite present in CS500 was consumed during the reaction and its nodule and nanocrystalline form potentially influenced its reactivity. In addition, the iron oxide contained in the tetrahedral sites participated in the precipitation. The consumption of the Fe-rich phases was in line with the formation of iron phosphate. The first halo was centered around 12° 2θ, where many of the reflections of iron phosphate were also observed. For that reason, it could be related to the amorphous iron phosphate formed during the reaction. The second hump was observed between 22 and 37° 2θ and corresponded to the formation of an amorphous structure [15]. This broad diffuse halo could have been due to the superposition of a diffuse halo characterizing the residual iron phase from the CS500 and another halo, which characterized the newly formed amorphous phase. Since iron phosphate was the main crystalline component that formed, it seemed logical that the newly formed amorphous phase contained less iron than before. Originally (before thermal treatment), this amorphous phase had a fayalitic composition, and the thermal treatment already consumed some of the Fe present in it to form iron oxides. Therefore, the remaining amorphous phase was enriched in Si, and thus was a silicate with a low Fe content. The XRD analysis alone could not distinguish the chemical element environment of amorphous substances. Therefore, the amorphous substances in PC were further studied by the FTIR analysis.

From this section, we concluded that the amorphous fraction of the slag decreased, and the crystalline features ascribed to hematite (Fe_2_O_3_), hercynite (FeAl_2_O_4_), and magnetite appeared or became more prominent as a result of the partial oxidation of fayalite at 500 °C, as predicted by Equation (1) (Section 3.1). Most of the crystalline fractions reacted with phosphoric acid to form iron phosphate but there was a large amorphous fraction that remained.

### 3.3. FTIR Analysis of the CS, CS500, and PC Samples

**Figure 5:**
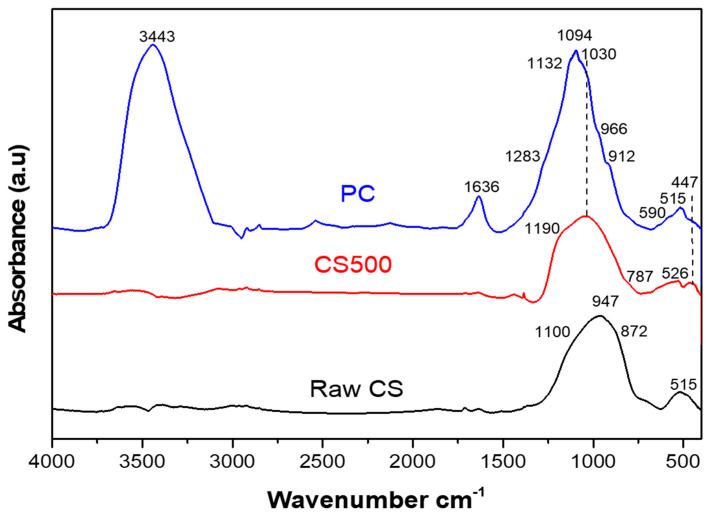
Infrared spectra of the CS, CS500, and PC samples.

**Figure 6:**
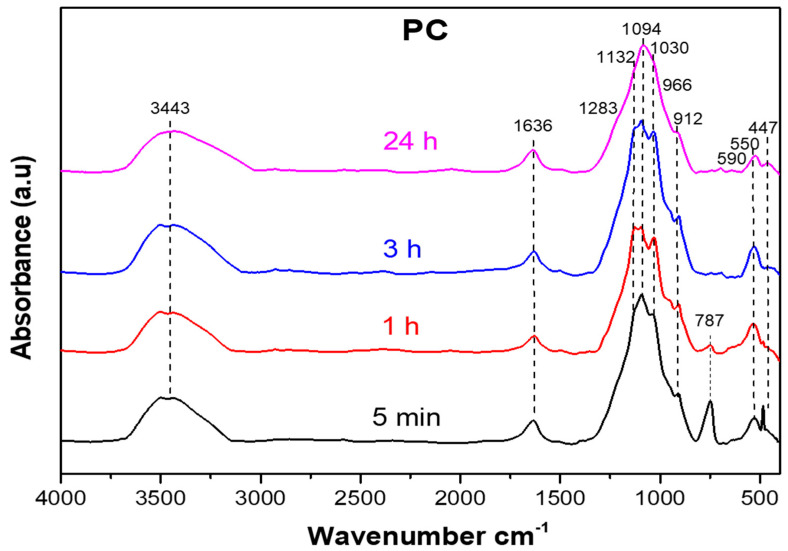
Infrared spectra of the PC samples at different curing times (5 min, 1 h, 3 h, and 24 h) at 20 °C. At the beginning, the spectrum was dominated by the absorptions from phosphoric acid. After 24 h, the spectrum was almost identical to one of a post-cured PC sample.

The IR curves of the CS500 and PC samples displayed a significant difference compared to that of the raw CS starting powder (Figure 5). In order to determine the chemical evolution with respect to the curing time, infrared spectroscopy was performed on the phosphate cementitious mixture during the first 24 h. The different FTIR spectra are shown in Figure 6.

The spectrum of untreated slag (CS) exhibited the typical characteristic peaks of fayalite slags, which were attributed to the stretching and bending modes of the SiO_4_ tetrahedra in fayalite [37]. A series of absorption bands was observed starting at 515 cm^−1^ corresponding to SiO_4_ symmetric bending in fayalite; a shoulder at 827 cm^−1^ corresponding to the Si–O symmetric stretching vibrations of the v1 mode; 872 and 947 cm^−1^ corresponding to the Si–O asymmetric stretching vibrations of the v3 mode; and 1100 cm^−1^ corresponding to amorphous SiO_2_ [15,38,39]. Finally, in total agreement with Onisei et al. [38], the glassy phase, which was the main slag constituent, was also expected to have a broad contribution in the 1250–750 cm^−1^ area. As was understood by Nikolov et al. [15] using Mössbauer spectroscopy, the primary constituents of this amorphous fraction were Fe^2+^ ions in the structure of the mineral fayalite (Fe_2_SiO_4_).

The CS500 spectrum showed a shift of the bands and a change in their intensity, indicating a structural change in the CS500 sample. This could be explained by the effect of the heat treatment at 500 °C on the slag structure that led to a significant modification in phase composition and the appearance of new crystalline phases. Indeed, the absence of the fayalite characteristic bands (515, 872 and 947 cm^−1^) and the shift of the most intense band towards higher wavelengths was associated with the oxidation of Fe^2+^ in the raw CS into Fe^3+^ in the CS500 [38]. The band at 1030 cm^−1^ was attributed to the mode of vibration of the Fe^3+^–O–Si bond, as was reported by Ponomar et al. [40], and indicated the formation of a new phase. The presence of the bands located at 1190 cm^−1^ and at 447 cm^−1^ could be attributed to the different modes of vibration of the Si–O bond [34]. The broad absorption in the wavenumber range from 1200–1000 cm^−1^ was characteristic of amorphous SiO_2_, which was caused by the asymmetric stretching vibration of the Si–O bond in SiO_2_ [41]. This silica was formed upon the extraction of Fe for the formation of the iron oxide phases. Moreover, the bands positioned at 787 cm^−1^ and 550 cm^−1^ were assigned to the iron oxide and hematite, respectively [34,42]. In line with Mihailova et al. [39], the change observed in the CS500 was typical of the transition from an amorphous to a crystal state and was related to the decomposition of fayalite during the thermal treatment of the copper slag. Moreover, the band widening in the 1250–750 cm^−1^ region, after heat treatment, was due to an overlap between the bands of the new structure and the bands associated with the undecomposed residual fayalite, as observed through the XRD analysis. The CS500, thus, consisted of iron oxide, mainly hematite, the remaining fayalite, and a silicate network with a reduced amount of Fe, where the other elements, such as Al and Ca, were still present.

The spectrum of the phosphate-activated cement (PC) showed two bands located in the high frequency zone (between 4000 cm^−1^ and 1600 cm^−1^): one at 3443 cm^−1^ and the other at 1636 cm^−1^, which are attributed to the stretching and deformation vibrations of free water, respectively. The appearance of these bands was due to the addition of water during the synthesis of the PC. The amount of water, however, decreased during the curing time due to evaporation. There were no sharp absorptions around 3300 cm^−1^, so no crystalline hydrated compounds such as hydrated iron phosphates were formed. Therefore, these hydrated compounds were amorphous, as shown by the broad bands that could not be distinguished from the free water that was present.

In the low frequency region (between 1200 cm^−1^ and 400 cm^−1^), the 447 cm^−1^ band linked to a bending mode of Si–O decreased or even disappeared in the FTIR spectra of the PC compared to the CS500, showing the consumption of the reagents during the reaction while the band at 550 cm^−1^ remained. The broad band observed at 1250–750 cm^−1^ in the CS500 spectrum shifted after the reaction to the 1400–800 cm^−1^ range, with a peak shift from 1030 cm^−1^ to 1094 cm^−1^. This shift was more difficult to interpret because, on one hand, the iron phosphate was formed from hematite, while on the other hand Fe^2+^ as well as Ca and Al could still be extracted from the remaining silicate, leaving a silicate network behind with absorptions in the same range. The oxidation of the residual Fe^2+^ in the CS500 to Fe^3+^ in the binder and iron agglutination at the nanoscale [21] could in part explain this shift to more Si–O–Si bonds and fewer Si–O–T bonds, with T being mainly Fe, Al, and Ca [17]. In addition, according to Nikolov et al. [15], the occurrence of the intense absorption within this spectral range strongly suggested the formation of a new mineral phase. The position of its maximum and the presence of a shoulder at 1283 cm^−1^ were within the range of the P–O stretching vibrations of the phosphates, silicate–phosphate glasses [42], and silica, which remained after the extraction of the cations. Moreover, in agreement with Abdelghany et al. [43], the band located at 1132 cm^−1^ in the PC spectra was attributed to the asymmetric stretch of P=O. Similarly, in agreement with Katsiki et al. [44], the bands observed at 966 cm^−1^ and 912 cm^−1^ were ascribed to the asymmetric stretching (P–O) and the vibration (P–OH) in aluminum phosphate, respectively. On the other hand, in the PC spectra (Figure 6), the chemical changes were apparent due to the presence of broad bands and a low intensity centered at 1094 cm^−1^ and 447 cm^−1^, which was likely ascribed to the (Si–O) bonds. The reduction in these bands constituted an indicator of the consumption of reagents and of the solidification of the material formed. The precursor was consumed during the dissolution–precipitation reaction from the PC^5min^ to PC^24h^. Consequently, the PC material contained an increasing degree of precipitation products as a function of the curing time. However, the persistence of the band at 550 cm^−1^ was good proof that part of the trivalent iron (Fe^3+^) form of the CS500 persisted in the PC structure. However, the small amount and poor ordering of hematite in the PC and the overlap with the other crystalline phases could be the reason for the absence of its characteristic reflections in the XRD pattern of the PC. This incomplete reaction could have been due to the low solubility of the trivalent metal oxides in an acidic environment.

The similarity of the PC sample spectra after a 24 h curing time and after post-curing indicated the fast kinetics of the reaction at an early stage. The disappearance in the PC^3h^ spectrum of the band at 787 cm^−1^ observed in the PC^5min^ and PC^1h^ spectra proved that all the Fe in this environment (iron oxide) contributed to the polymerization reaction and was integrated in the three-dimensional structure [44]. The characteristic bands around 1132 and 1094 cm^−1^ comprised a single broad band at 1094 cm^−1^ after 24 h of curing. The broadening of the characteristic bands was due to the hardening and the different chemical environments that were frozen during hardening, for instance the bond angles that were not always the same in the tetrahedra of FeO_4_^5−^. This fast evolution of the chemistry, however, was not in line with the poor mechanical strength after hardening for 24 h at 20 °C. The heat evolution during the chemical reaction will, thus, be studied to get a better view on how these chemical processes continue.

The IR analysis confirmed that the Fe-containing phases were consumed during the reaction, except for some Fe^3+^ which was difficult to observe using XRD. IR was also more suited for monitoring the amorphous structures, but the amorphous silica formed according to Equation (1) overlapped with the phosphate, making further elucidation cumbersome. The chemistry changed in the first 24 h at room temperature with little changes afterwards, although at that moment the sample had only just sets, and a large part of the strength still needed to be built up. Isothermal calorimetry could monitor the slow processes for a longer time, as will be shown in the next section.

### 3.4. Isothermal Microcalorimetry

**Figure 7:**
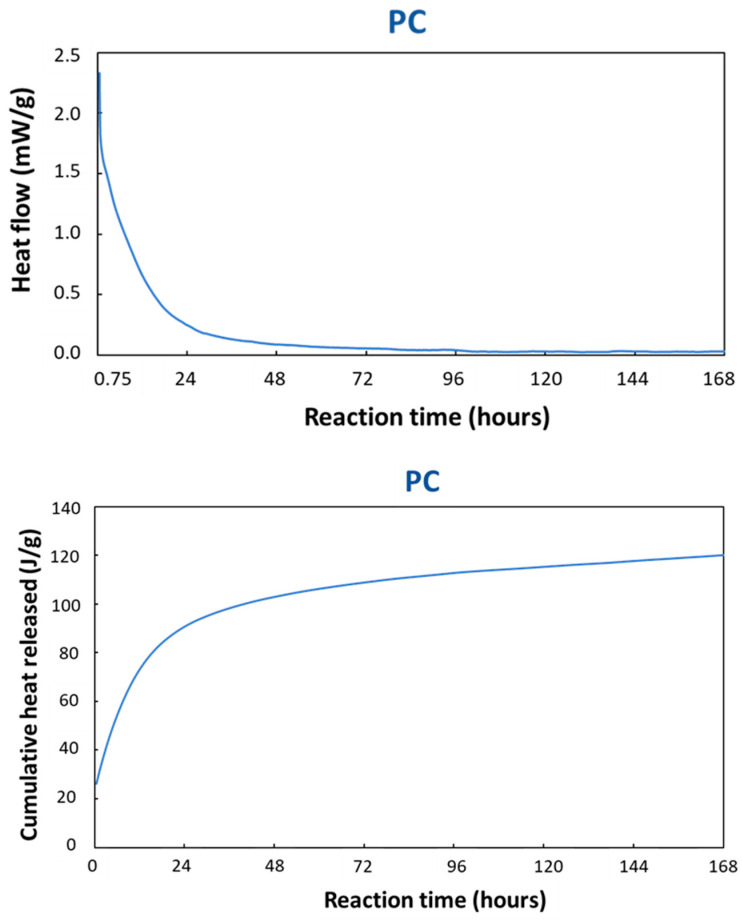
Normalized heat flow and the cumulative heat released for the PC at 20 °C.

**Figure 8:**
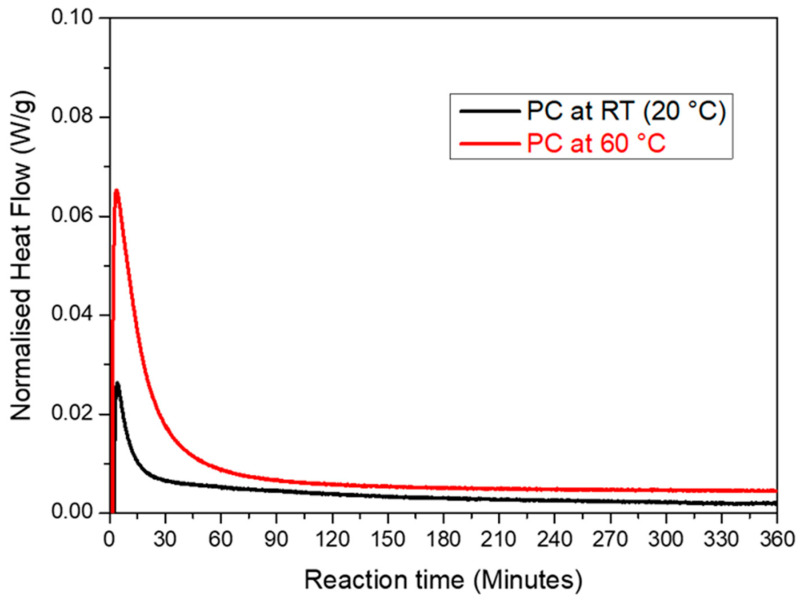
Comparison of the rate of the reaction of the PC sample isothermally at 20 °C and 60 °C during the first 6 h of the experiment.

In order to determine the stoichiometry of the PC reaction and to get an idea for the reaction kinetics, isothermal calorimetry was employed on TAM Air (Figure 7) and DSC (Figure 8). The TAM Air measurement carried out on a PC sample is presented in Figure 7. The first 45 min of the reaction were not monitored due to the time expended in the sample preparation and the time required to stabilize the instrument at the equilibrium temperature. The heat flow data was continuously recorded over a period of 7 days throughout the reaction. These heat flow values were then normalized by dividing them by the total weight of the paste, allowing for a more accurate assessment and comparison of the thermal characteristics of the reaction [44,45]. The normalized and cumulative heat flow showed an important exothermic peak at the beginning of the measurements corresponding to a rapid dissolution process of the reactive particles in the acid medium. Indeed, at the first contact of the copper slag with the phosphoric acid solution, the chemical reaction started immediately and released a large amount of heat [46]. After the first quick dissolution, the reaction rate, which was proportional to the heat flow, decreased gradually, which was almost comparable to a first order reaction. The setting of the binder occurred gradually after about 24 h of reaction without a specific change in the heat flow. The reaction continued releasing heat over a large span of time while the specimen hardened. This can be better observed on the cumulative heat diagram shown in Figure 7. This was the total heat released during the reaction until a certain moment. After one day, the heat released was approx. 90 J/g, while after 7 days it was approx. 110 J/g. However, the reaction rate became very small, and the heat flow signal disappeared in the baseline. The reaction rate of the raw CS with phosphoric acid was extremely high, taking only minutes to set. Thus, it was impossible to measure it using TAM Air. However, setting was reduced to a few hours after the CS was thermally treated at 500 °C (Figure 7). Thus, due to the thermal treatment and structural changes of the CS, the CS500 had a more reduced the rate of dissolution. The reaction proceeded gradually, but it was not possible to distinguish the different steps.

The isothermal DSC measurements were employed to explore the impact of the curing temperature on the heat flow variations during the precipitation process for the PC sample at two curing temperatures (20 °C and 60 °C). The advantage of DSC is that the overshoot at the beginning of the experiment was limited to a few minutes compared to 45 min for TAM AIR. The disadvantage was the lower stability of the baseline, thus the measuring time for an isothermal experiment was limited to a few hours. At both temperatures, a significant exotherm was observed during the first 5 min. This was attributed to the dissolution of the various precursors [47]. Afterwards, the reaction rate decreased gradually. The reaction rate decreased with a factor of approx. five after 45 min. This was the time when the data for the isothermal calorimetry (Figure 7) began to be measured. Using isothermal calorimetry, a large part of the reaction could not be recorded at the start of the reaction. These experiments showed the important influence of the curing temperature as a kinetic agent for accelerating the precipitation reaction. The heat released during the reaction was approximately 9.4 J/g at 20 °C and 73 J/g at 60 °C. This was much less than what was shown in Figure 7, but it was also measured over a much shorter time with a very small amount. In addition, it indicated that if a large sample was immediately heated to 60 °C, it could overheat because a lot of heat would be released in the first few minutes and the sample would heat up too quickly, causing thermal stress and cracks in the sample. The reaction heat after 6 h was much higher at 60 °C than at 20 °C, which also explained why the sample only started to set after 24 h at 20 °C. This was why the curing procedure chosen in this work was first cured at 20 °C (room temperature) followed by post-curing at 60 °C.

Isothermal calorimetry showed that the reaction started immediately after mixing, with the largest reaction rate in the beginning. A big portion of the reaction heat was released in the first 24 h, i.e., before the setting started at room temperature. The dissolution went smoothly over into setting. At 20 °C, the reaction took a long time, thus post-curing at 60 °C considerably sped up the hardening. The mechanical properties could, thus, be expected to change over a long period of time, which will be investigated in the next section.

### 3.5. Mechanical Properties

**Figure 9:**
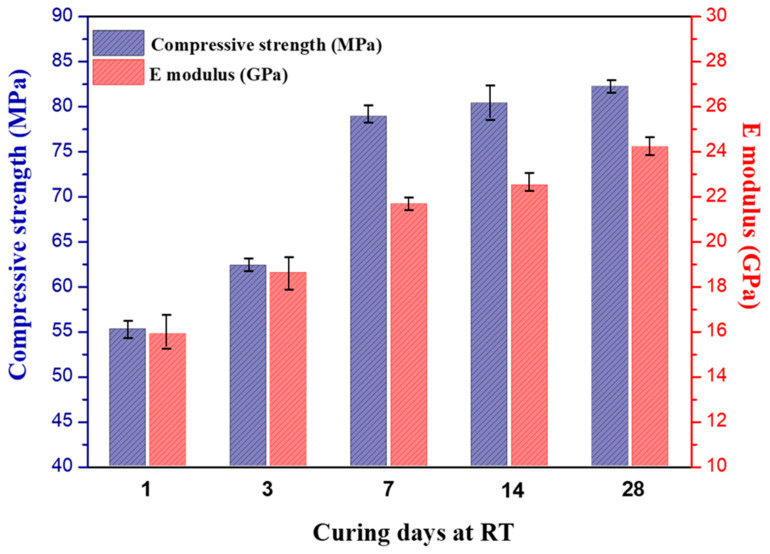
Evolution of the compressive strength and elastic modulus of the PC as a function of the curing time (1 day: after post-cure at 60 °C and demolding).

The mechanical properties of the phosphate cementitious materials (PC) at different curing days after post-curing are presented in Figure 9. The first observation was that the compressive strength and E-modulus proportionally increased from 1 to 28 curing days even after post-curing. The PC mechanical resistance increased from 55.2 MPa after 1 curing day to 82.5 MPa after 28 curing days. Similarly, the E-modulus rose from 15.9 GPa after 1 curing day to 24.2 GPa after 28 curing days. As shown in Figure 9, the increase in the mechanical properties of the PC was higher compared to the previous studies [15,28]. These authors reported that the compressive strength of the raw CS-based phosphate cements was equal to 17 MPa. The second significant observation pertained to the rate of increase in the mechanical resistance. There was a slight increase in the mechanical resistance of 4.8% between 7 and 28 curing days. This increase could be explained by the faster kinetics of the reaction in the PC samples. In addition, in line with Louati et al. [48], the densification of the phosphate cements was improved as the curing age increased. This finding proved that the rapid setting and strength gain resulted from a better dissolution of the heated copper slag, leading to improved precipitation and hardening and resulting in an improvement in the mechanical properties. Moreover, the formed cementitious material appeared as a dense composite, as indicated by the SEM results (the first precipitates were probably rather amorphous and crystallized over time). The crystalline phases were also likely to have contributed to the improvement of the mechanical properties of the obtained material [49]. This finding correlated with the results reported through XRD. Therefore, the obtained phosphate cementitious material would be a composite consisting of amorphous phases and crystalline phases. Additionally, the remaining silicate was amorphous but did not function as a binder [50]. The heated copper slag-based phosphate cements led to a good strength build up and even a higher final strength compared to the literature. The compressive strength and the Young’s modulus of the PC increased with the increase in the curing time, and the increase presented a similar trend. The strength growth rate during 0–7 days was greater than that of 7–28 days. During 0–28 days of curing, the mechanical properties increased continuously, indicating that the precipitation of the copper slag-based phosphate cement was a long-term process and that post-curing at 60 °C for 24 h was not enough to reach a 100% conversion.

Although at room temperature the reaction was slow, the compressive strength was approx. 55 MPa after 24 h of curing at 20 °C and 24 h post-curing at 60 °C. It slowly increased further over time to 80 MPa after one month, showing that 24 h post-curing at 60 °C was not enough to reach full conversion. In the next and last section, the microstructure of the phosphate cement will be investigated using SEM with the aim of trying to understand how the slag particles changed during the reaction, how the binder adhered to the remaining slag particles, and if the binder could fill the holes between the original slag particles to make a more or less dense material.

### 3.6. Morphology and Microstructure of the CS, CS500, and PC Samples

**Figure 10:**
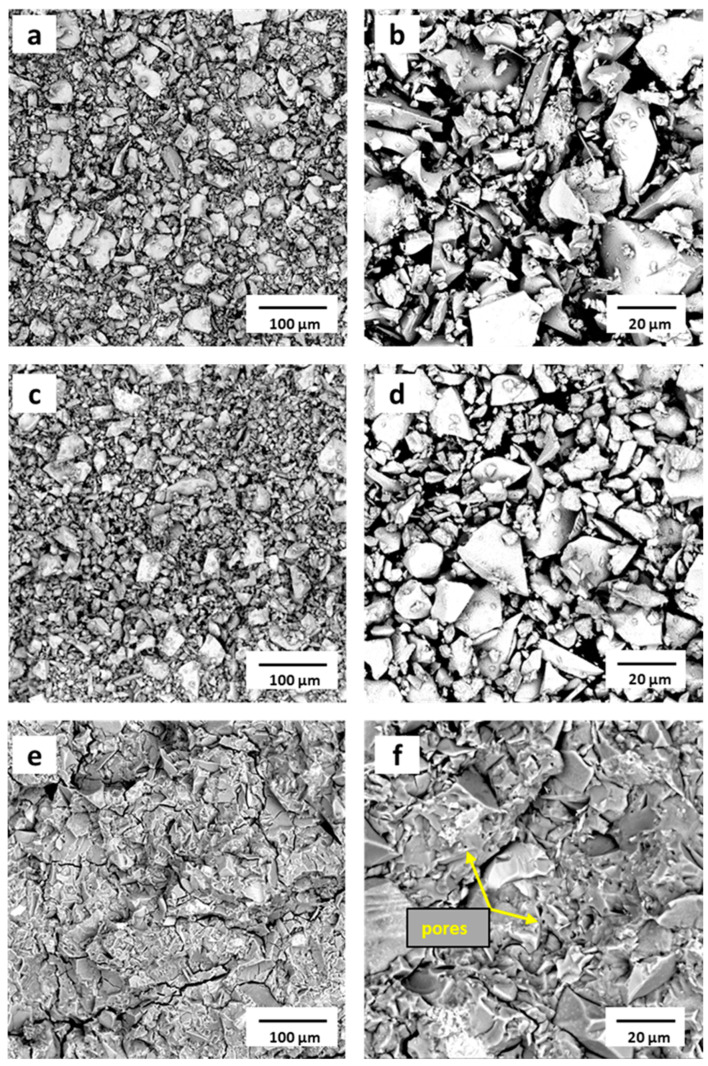
SEM analysis of (**a**,**b**) the raw CS; (**c**,**d**) CS500, and (**e**,**f**) PC samples (fractured surface after the compressive strength test).

As can be seen from the SEM micrographs, there was a similarity in the morphology of the raw copper slag CS (Figure 10a,b) and the heat treated CS500 (Figure 10c,d) samples. The particle size distribution of the copper slag particles varied unevenly, ranging from a few micrometers to several tens of micrometers, as shown in the particle size distribution (Figure 1). Regardless of whether the particles were large or small, the slag exhibited a smooth surface and dense structure in agreement with the literature [41]. Indeed, the morphology of the raw CS was composed of non-spherical glassy microstructures with irregular shapes [51]. The observed angular particles with a rough texture and irregular shape were attributed to the presence of FeO, although it could be observed for more glasses than were crushed. This irregular shape of the CS was preserved in the CS500, as shown in Figure 10c,d. This result was in line with several studies that reported that the thermal treatment at 500 °C induced a structural transformation but did not cause any change in morphology. The small difference in the particle size distribution (maximum shifts from 17 to 20 microns and the disappearance of small particles below 7 microns) was not easily observed in the pictures. The reason why they were not detected was probably because they were stuck to larger particles. The physical characteristics of the CS500 influenced the mechanical properties of the prepared phosphate binder positively.

The scanning micrograph images of the phosphate-activated material (PC) after the compressive strength test are plotted in Figure 10e,f. They highlighted a dense phase characteristic of the binder. The newly formed phosphate cement network was homogeneous and compacted, and it was difficult to distinguish between the matrix and remaining slag particles [52]. The presence of some granular structures visible at 20 µm proved that the iron compound did not fully participate in the matrix bonding for the synthesis of the network. This finding correlated with the XRD and FTIR analyses of the PC sample. However, the binder structure contained cracks, which were probably formed through the compressive strength measurements. The smooth surfaces also contained very small pores of approx. 1 micron. One reason could have been due to entrapped air during mixing. Another reason could have been that the setting process was an exothermic reaction resulting in the generation of heat (free water evaporation). These pores were commonly observed in chemically bonded phosphate cements and were prevalent in this material too [11].

The influence of the heat treatment of the fayalitic copper slag on the properties, microstructures, and structures of the copper slag and phosphate cements led to the following conclusions.

(1) The use of heat treatment for 2 h at a temperature of 500 °C led to changes in the phase composition following several processes. The most important ones were the oxidation of Fe^2+^ into Fe^3+^, involving the formation of hematite leaving a silicate phase with a reduced iron content and some Al and Ca behind. From the outside, the particles still looked the same, thus a more in-depth study would be needed to get a better insight into the rearrangement of the phases with the particles.
(2) The XRD and FTIR analyses revealed that the heating at 500 °C caused a partial transition. It transformed fayalite, the dominant crystal phase in the raw CS, into hematite, magnetite, and silica. Magnetite underwent a complex oxidation process, resulting in the formation of the equilibrium hematite phase Fe_2_O_3_.
(3) The thermal treatment reduced the reactivity of the copper slag with phosphoric acid, giving sufficient time for the mixing and casting of the paste, yielding a compact phosphate as observed using SEM with a high compressive strength. The phosphate cement was most probably built up of a binder phase, consisting of phosphate products, mainly iron phosphate that glued the remaining slag particles together. The adhesion seemed to be very good, and it was difficult to distinguish the remaining slag from the binder. EDX mapping could help to elucidate the complex structure of the material.
(4) The compressive strength of the newly developed cement was 78.9 MPa after 7 curing days, reaching up to 82.5 MPa after 28 curing days. The curing procedure still needs to be optimized since a schedule of 24 h at 20 °C followed by a post-cure of 24 h at 60 °C rendered only 70% of the obtained compressive strength after an additional 28 days of hardening at room temperature.

## 4. Conclusions

The phosphate-based cement was prepared reacting heated copper slag (CS500) with phosphate acid solution. The exposure of the copper slag to heat treatment at 500 °C induced a partial structural transformation. At this high temperature, all of the divalent iron oxide contained in the slag underwent oxidation, resulting in the formation of hematite and some other phases in smaller amounts next to the remaining silicate, as shown by the XRD and IR. This treatment reduced the reactivity of the copper slag and extended the setting time when exposed to phosphoric acid, resulting in a dense phosphate-based cementitious material with exceptional mechanical properties. The ease of formation, slower setting, and high mechanical strength of copper slag-based phosphate cements make them promising candidates for various special applications, such as the production of textile-reinforced cementitious composites (TRC).

More work is, however, needed to further elucidate the relationships between the processing, structure, and properties of the material. The influence of the temperature of the thermal treatment, and thus the amount of oxidation, on the reactivity and mechanical properties will be studied and published in the next paper. EDX mapping can reveal the structure of the cement, namely it can show if remaining slag particles exist or if the matrix is homogeneous. The cure procedure needs further optimization. For instance, if the material would be used as matrix for high-volume fraction composites that are cured after shaping, similar to a composite with a thermoset matrix, the producer would need to minimize the time before unmolding, requiring more profound knowledge on the relationship between the time and temperature of the curing and the strength development.

The findings of this work would be expected to help industrials choose the most appropriate precursor and production method for the synthesis of phosphate cements, and thus reduce the huge amounts of fayalite slag generated for the last decades as a result of copper production. Equally, this work would contribute to the production of phosphate products with better mechanical characteristics. The thermal treatment process of copper slag is very useful for producing phosphate cementitious materials with a high performance.

## Figures and Tables

**Table 1:** Normalized chemical composition in wt% of the raw CS determined by the XRF analyses. * Includes other small amounts below 1 wt%.

Oxide	FeO	Fe_2_O_3_	SiO_2_	Al_2_O_3_	ZnO	CaO	MgO	Cr_2_O_3_	P_2_O_5_	CuO	MnO	SO_3_	TiO_2_	K_2_O	Others *
Raw CS	47.1	3.34	27.0	8.48	4.14	3.33	0.87	1.47	0.68	0.67	0.84	0.73	0.35	0.16	0.85

## Data Availability

The data presented in this study are available upon request from the corresponding authors.
